# Is Chronic Inflammation a Possible Cause of Obesity-Related Depression?

**DOI:** 10.1155/2009/439107

**Published:** 2009-06-29

**Authors:** Magdalena Olszanecka-Glinianowicz, Barbara Zahorska-Markiewicz, Piotr Kocełak, Joanna Janowska, Elżbieta Semik-Grabarczyk, Tomasz Wikarek, Wojciech Gruszka, Piotr Dąbrowski

**Affiliations:** Department of Pathophysiology, Medical University of Silesia, 40-752 Katowice, Poland

## Abstract

Adult obesity has been associated with depression, especially in women. Whether depression leads to obesity or obesity causes depression is unclear. Chronic inflammation is observed in obesity and depression. In 63 obese women without additional diseases depression level was assessed with the Beck's questionnaire. After evaluation of depression level study group was divided into groups according to the mood status (A—without depression, B—mild depression, and C—severe depression), and serum concentration of TNF-*α*, sTNFs, leptin, and IL-6 were measured by ELISA. No differences in age, body mass, BMI, and body composition were observed in study groups. We did not observe differences of serum concentrations of TNF-*α*, sTNFRs, leptin, and IL-6 between subgroup A and subgroups B and C. It seems that circulating adipokines did not exert influence on depression levels in obese women.

## 1. Introduction

The prevalence of both obesity and depression has been increasing for decades [1, 2]. There has been evidence for association of both disorders with increased risk of mortality [3], coronary heart disease [4], hypertension [5], and diabetes [6]. It seems that there may be a link between obesity and depression.

Adulthood obesity has been associated with depression, especially in women [7]. This association seems to be clearer in subjects with central obesity [8]. Central obesity is a greater risk factor for metabolic complications and cardiovascular disease than peripheral obesity [9]. Independent increase of risk for coronary heart disease in depressive mood patients may be explained by a positive association between depression and visceral adipose tissue [10]. Whether depression leads to obesity or obesity causes depression is unclear.

It has been shown that depression and obesity share neurobiological pathophysiological mechanisms [11]. Disturbances in serotonin release may be connected with recurrent increase of carbohydrate-rich foods consumption and inability to physical activity [11]. Other evidences show a link between depressive syndromes, and distorted hypothalamic-pituitary-adrenal (HPA) axis function; it seems that chronic excessive action of cortisol is responsible for accumulating fat [12]. Stress is also a powerful factor activating HPA axis [12]. It seems that brain reward circuity plays the central role in the activation of stress-induced food intake. Either highly palatable food or activation of HPA releases opioids attenuating detrimental effect of stress response by inhibiting HPA [12]. Chronic stress may lead to dysregulation of balanced system, increase of calories intake, and visceral fat accumulation [12] with such consequences as hypertension, diabetes, and dyslipidemia [13]. New concept of depression assumes this disorder as a chronic endogenous stress [14].

It seems that there can be a theoretical link between early onset stress and morphological changes of neuroendocrine axis that can cause both obesity and depression [15]. Moreover, unsuccesful attempts at food restrictions as a stress factor may cause increase of the value of consumed palatable food [12]. Increased concentration of cortisol increases the reward value of food via increased action of peptide mediators [12]. Mediators enhancing appetite include neuropeptide Y (NPY), leptin, and insulin [12]. Alterations in modulation mechanisms may lead to compulsive overeating and seem to be responsible for ineffective treatment of obesity in long term [12]. Disturbation of adrenal hormones metabolism seen in depression and disturbances in carbohydrates and lipid metabolism may lead to the development of metabolic syndrome, a factor promoting atherosclerosis and development of cardiovascular disease and increase of mortality [16]. 

Current study on pathopysiology of depression revealed that immune activation observed in obesity may be one cause of development of depression [17].

 The aim of the study was to assess serum concentrations of TNF-*α*, TNF soluble receptors, leptin, and interleukin-6 (IL-6 ) in obese subjects with and without depression.

## 2. Material and Methods

The study was carried out on 63 obese women—weight 98.2 ± 16.4 kg, aged 55.6 ± 7.5 years. Their body mass index (BMI) was 37.9 ± 5.5 kg/m^2^. All subjects were diagnosed as having simple obesity with no concomitant diseases and without any pharmacological treatment. The weight of our obese patients was stable at the enrollment—both patients with the sudden lost and patients with the sudden increase of weight were excluded from the observation. Their case histories of obesity have lasted for at least some years. 

The exclusion criteria included evidence of present or recent (preceding 3 months) infectious disease, fever, and drug therapy. 

The study was approved by the local ethics committee. All subjects gave their informed consent for the study.

The body weight and height were measured, and body mass index (BMI) was calculated. Body composition was assessed by impedance analysis using Bodystat analyzer. 

The Beck's questionnaire was used to assess depression level. After evaluation of depression level study group was differentiated into subgroups: A—subjects without depression (6.1 ± 3.0 points) *n* = 24, B—subjects with mild depression *n* = 33, and C—subjects with severe depression *n* = 5.

The determinations of TNF-*α* and sTNFRs, leptin, and IL-6 in the serum were carried out by enzyme-linked immunosorbent assay. 6–8 mL samples of venous blood were collected from each subject in the morning, after an overnight fast between 8-9 a.m. After clot formation, the samples were centrifuged (1000 g) at a room temperature for 10 minutes. The obtained serum was drawn into a few plastic vials and stored at −80°C until the time of assay. 

TNF-*α* and soluble forms of both TNF-*α* receptors (sTNF-R1 and sTNF-R2) were measured using a commercially available highly sensitive ELISA kits (Genzyme Diagnostics, Cambridge, USA). 

The minimum detectable concentration of TNF-*α* is typically less than 0.18 pg/mL. Mean intraassay coefficient of variance was 14.4%, and mean interassay coefficient of variance was 18.7%. 

The minimum detectable concentration of sTNF-R1 is typically less than 3.0 pg/mL. Mean intraassay coefficient of variance was 2.9%, and mean interassay coefficient of variance was 3.7%. 

The minimum detectable concentration of sTNF-R2 is typically less than 1.0 pg/mL. Mean intraassay coefficient of variance was 2.5%, and mean interassay coefficient of variance was 3.5%. 

 Leptin was measured using a commercially available highly sensitive ELISA kits (BioVendor Laboratory Medicine Inc., Czech Republic).

The minimum detectable concentration of leptin is typically less than 0.17 ng/mL. Mean intraassay coefficient of variance was 5.4%, and mean interassay coefficient of variance was 7.8%. 

IL-6 was measured using a commercially available highly sensitive ELISA kits (Diagnostic Products Corporation, USA).

The minimum detectable concentration of IL-6 is typically less than 1.0 pg/mL. Mean intraassay coefficient of variance was 10.0%, and mean interassay coefficient of variance was 10.0%. 

 All text and table values are expressed as means ± SD. The U-Manna-Withney's test was used to compare study groups. The relationships between study parameters were examined by Spearman's correlation analysis. A value *P* < .05 was considered statistically significant.

## 3. Results

The characteristics of study subgroups are presented in Table 1.

There were no differences of age, body mass, BMI, and body composition between study subgroups (Table 1).

We did not observe differences of serum concentrations of TNF-*α*, sTNFRs, leptin, and IL-6 between subgroup A and subgroups B and C (Table 2).

We found positive correlations between body mass and serum concentration of TNF and sTNFR1 (*r* = 0.4802, *P* = .032 and *r* = 0.4518, *P* = .046, resp.) and between BMI and sTNFR 1 concentration and (*r* = 0.4451, *P* = .049) in subgroup A.

Positive correlations between body mass and serum concentrations of sTNFR 2 and leptin were found in subgroup B (*r* = 0.3354, *P* = .03 and *r* = 0.3046, *P* = .05, resp.) and between BMI and serum concentrations of sTNFR 2 and leptin (*r* = 0.3446, *P* = .025 and *r* = 0.4004, *P* = .009, resp.). 

When the depressive subjects were divided into pre- and postmenopausal, no differences in weight, body composition, and waist circumference were observed. There were also no differences between study cytokines. In the nondepressive subjects there were also no differences in study parameters between pre- and postmenopausal women.

## 4. Discussion

In some epidemiological studies the relationship between obesity and depression was established [18]. It suggests that depression may be a trigger for development of visceral obesity. It has been postulated that in subjects with depressive syndrome accumulation of visceral fat occurs, which promotes inflammatory response [19]. In the increased release of cytokines in depressive patients at least two mechanisms are involved: expanded release of cytokines from adipose tissue and upregulation of interleukin release by leptin [19]. 

In our recent study [20–22] we observed increased serum concentrations of TNF-*α* and IL-6 and other authors [23] revealed increased serum concentration of leptin in obese subjects. Increased concentrations of cytokines were explained as a result of increased visceral adiposity in depressive patients [19]. Cytokines are responsible for increased risk for coronary heart disease and diabetes [24, 25]. As it was described above these medical conditions may be factors of depression enhancing. 

 In the present study we did not observe differences of serum concentrations of TNF-*α*, IL-6, and leptin between depressive and nondepressive patients with obesity. These results are contradictory to results obtained by Miller's et al. [26], who observed elevated concentrations of IL-6 in depressive patients. However, patients with depressive syndrome studied by these authors had greater body mass than controls, which could, in part, be responsible for increased concentration of cytokine. Recent study revealed that about 30% of IL-6 is produced in adipose tissue [27]. In other papers published by Miller's et al. [19] no differences in cytokines such as IL-6 and leptin were detected in young obese persons with and without depressive syndrome. 

 Yang et al. [28] found higher concentrations of serum level of IL-6, TNF-*α*, and lower level of leptin in subjects with depression in comparison to controls without depressive disorder; moreover, higher levels of leptin were observed in both healthy and depressive women than in men. In these studies BMI was not considerd as a factor that may influence cytokines levels.

Results obtained by Lespérance's et al. study [29] are in accordance with ours, because they did not also reveal differences in circulating level of interleukin-6 between subjects with and without depression and association between concentration of IL-6 and the level of depression. 

On the other hand, Levine et al. [30] revealed increased level of interleukin-1 beta, interleukin-6 were detected in cerebrospinal fluid in acute diagnosed depression, but there were no differences in TNF-*α* concentrations between depressive patients and healthy controls. Unfortunately, on the basis of these interesting results, it is difficult to explain if increased concentrations of these cytokines are primary or secondary to depression. 

Further studies are necessary to clarify the association between immune activation and depression. However, taking into account that obesity often accompanies depression and the obesity itself is a chronic inflammation process, nutrition status of study subjects is very important while analyzing results from studies on depressive patients. This hypothesis is supported by the observed, in our study, significant positive correlations between BMI and serum concentrations of sTNFR 2 and BMI and leptin in depressive, obese subject, which suggest that the level of obesity is more associated with immune activation than depression.

## 5. Conclusions

It seems that circulating adipokines did not exert influence on depression levels in obese women.

## Figures and Tables

**Table 1:** Patients' characteristics.

		A	B	C
N		24	33	5
Age	(year)	53.7 ± 6.0	57.0 ± 8.2	55.6 ± 8.5
Body mass	(kg)	96.8 ± 14.5	98.8 ± 16.2	101.2 ± 27.9
BMI	(kg/m^2^)	37.1 ± 4.6	38.3 ± 5.8	38.8 ± 8.3
Body fat	(kg)	48.2 ± 11.8	51.1 ± 12.9	51.6 ± 22.2
Body fat	(%)	49.3 ± 6.5	51.2 ± 7.0	49.5 ± 7.8
Fat-free mass	(kg)	48.6 ± 7.2	47.4 ± 7.6	49.5 ± 6.3
Fat-free mass	(%)	50.8 ± 6.4	48.8 ± 7.4	50.5 ± 7.6
Waist circumference	(cm)	102.0 ± 22.2	106.8 ± 11.4	110.0 ± 18.3

**Table 2:** Serum concentrations of TNF-*α*, soluble TNF receptors, leptin, and IL -6.

		A	B	C
TNF-*α*	(pg/mL)	5.9 ± 2.0	6.4 ± 2.0	4.9 ± 2.7
sTNFR1	(pg/mL)	1369.6 ± 528.3	1535.5 ± 556.38	1554.2 ± 674.8
sTNFR2	(pg/mL)	2179.6 ± 704.2	2259.7 ± 726.2	2323.3 ± 582.3
Leptin	(ng/mL)	34.0 ± 12.1	32.8 ± 18.1	32.4 ± 12.3
IL-6	(pg/mL)	10.1 ± 3.4	10.3 ± 3.3	8.7 ± 3.0

## References

[1] Flegal KM, Carroll MD, Ogden CL, Johnson CL (2002). Prevalence and trends in obesity among US adults, 1999-2000. *Journal of the American Medical Association*.

[2] Anderson RJ, Freedland KE, Clouse RE, Lustman PJ (2001). The prevalence of comorbid depression in adults with diabetes: a meta-analysis. *Diabetes Care*.

[3] Schulz R, Beach SR, Ives DG, Martire LM, Ariyo AA, Kop WJ (2000). Association between depression and mortality in older adults: the Cardiovascular Health study. *Archives of Internal Medicine*.

[4] Ferketich  AK, Schwartzbaum  JA, Frid  DJ, Moeschberger  ML (2000). Depression as an antecedent to heart disease among women and menin the NHANES 1 study. National Health and Nutrition Examination Survey. *Archives of Internal Medicine*.

[5] Davidson K, Jonas BS, Dixon KE, Markovitz JH (2000). Do depression symptoms predict early hypertension incidence in young adults in the CARDIA study? Coronary Artery Risk Development in Young Adults. *Archives of Internal Medicine*.

[6] Nichols GA, Brown JB (2003). Unadjusted and adjusted prevalence of diagnosed depression in type 2 diabetes. *Diabetes Care*.

[7] Roberts RE, Deleger S, Strawbridge WJ, Kaplan GA (2003). Prospective association between obesity and depression: evidence from the Alameda County Study. *International Journal of Obesity*.

[8] Roberts RE, Kaplan GA, Shema SJ, Strawbridge WJ (2000). Are the obese at greater risk for depression?. *American Journal of Epidemiology*.

[9] Rexrode KM, Buring JE, Manson JE (2001). Abdominal and total adiposity and risk of coronary heart disease in men. *International Journal of Obesity*.

[10] Lee ES, Kim YH, Beck S-H, Lee S, Oh SW (2005). Depressive mood and abdominal fat distribution in overweight premenopausal women. *Obesity Research*.

[11] Wurtman JJ (1993). Depression and weight gain: the serotonin connection. *Journal of Affective Disorders*.

[12] Adam TC, Epel ES (2007). Stress, eating and the reward system. *Physiology and Behavior*.

[13] Björntorp P, Rosmond R (2000). Neuroendocrine abnormalities in visceral obesity. *International Journal of Obesity*.

[14] De Kloet ER (2003). Hormones, brain and stress. *Endocrine Regulations*.

[15] Gohil BC, Rosenblum LA, Coplan JD, Kral JG (2001). Hypothalamic-pituitary-adrenal axis function and the metabolic syndrome X of obesity. *CNS Spectrums*.

[16] Petrlová B, Rosolova H, Hess Z, Podlipný J, Šimon J (2004). Depressive disorders and the metabolic syndrome of insulin resistance. *Seminars in Vascular Medicine*.

[17] Kim Y-K, Na K-S, Shin K-H, Jung H-Y, Choi S-H, Kim J-B (2007). Cytokine imbalance in the pathophysiology of major depressive disorder. *Progress in Neuro-Psychopharmacology and Biological Psychiatry*.

[18] Goodman E, Whitaker RC (2002). A prospective study of the role of depression in the development and persistence of adolescent obesity. *Pediatrics*.

[19] Miller GE, Freedland KE, Carney RM, Stetler CA, Banks WA (2003). Pathways linking depression, adiposity, and inflammatory markers in healthy young adults. *Brain, Behavior, and Immunity*.

[20] Zahorska-Markiewicz B, Janowska J, Olszanecka-Glinianowicz M, Zurakowski A (2000). Serum concentrations of TNF-*α* and soluble TNF-*α* receptors in obesity. *International Journal of Obesity*.

[21] Olszanecka-Glinianowicz M, Zahorska-Markiewicz B, Janowska J, Zurakowski A (2004). Serum concentrations of nitric oxide, tumor necrosis factor (TNF)-*α* and TNF soluble receptors in women with overweight and obesity. *Metabolism*.

[22] Olszanecka-Glinianowicz M, Zahorska-Markiewicz B, Janowska J (2006). The effect of weight loss on serum concentrations of nitric oxide, TNF-*α* and soluble TNF-*α* receptors. *Polish Journal of Endocrinology*.

[23] Considine RV, Sinha MK, Heiman ML (1996). Serum immunoreactive-leptin concentrations in normal-weight and obese humans. *The New England Journal of Medicine*.

[24] Sbarsi I, Falcone C, Boiocchi C, Campo I, Zorzetto M (2007). Inflammation and atherosclerosis: the role of TNF and TNF receptors polymorphisms in coronary artery disease. *International Journal of Immunopathology and Pharmacology*.

[25] Antoniades C, Tousoulis D, Marinou K, Papageorgiou N, Bosinakou E (2007). Effects of insulin dependence on inflammatory process, thrombotic mechanisms and endothelial function, in patients with type 2 diabetes mellitus and coronary atherosclerosis. *Clinical Cardiology*.

[26] Miller GE, Stetler CA, Carney RM, Freedland KE, Banks WA (2002). Clinical depression and inflammatory risk markers for coronary heart disease. *American Journal of Cardiology*.

[27] Bastard J-P, Maachi M, Lagathu C (2006). Recent advances in the relationship between obesity, inflammation, and insulin resistance. *European Cytokine Network*.

[28] Yang K, Xie G, Zhang Z (2007). Levels of serum interleukin (IL)-6, IL-1*β*, tumour necrosis factor-*α* and leptin and their correlation in depression. *Australian and New Zealand Journal of Psychiatry*.

[29] Lespérance F, Frasure-Smith N, Théroux P, Irwin M (2004). The association between major depression and levels of soluble intercellular adhesion molecule 1, interleukin-6, and C-reactive protein in patients with recent acute coronary syndromes. *American Journal of Psychiatry*.

[30] Levine J, Barak Y, Chengappa KNR, Rapoport A, Rebey M, Barak V (1999). Cerebrospinal cytokine levels in patients with acute depression. *Neuropsychobiology*.

